# ROC-Boosting: A Feature Selection Method for Health Identification Using Tongue Image

**DOI:** 10.1155/2015/362806

**Published:** 2015-10-12

**Authors:** Yan Cui, Shizhong Liao, Hongwu Wang

**Affiliations:** ^1^School of Computer Science and Technology, Tianjin University, 72 Weijin Road, Nankai District, Tianjin 300072, China; ^2^Department of Common Required Courses, Tianjin University of Traditional Chinese Medicine, 312 Anshanxi Road, Nankai District, Tianjin 300193, China; ^3^College of Traditional Chinese Medicine, Tianjin University of Traditional Chinese Medicine, 312 Anshanxi Road, Nankai District, Tianjin 300193, China

## Abstract

*Objective*. To select significant Haar-like features extracted from tongue images for health identification.* Materials and Methods*. 1,322 tongue cases were included in this study. Health information and tongue images of each case were collected. Cases were classified into the following groups: group containing 148 cases diagnosed as health; group containing 332 cases diagnosed as ill based on health information, even though tongue image is normal; and group containing 842 cases diagnosed as ill. Haar-like features were extracted from tongue images. Then, we proposed a new boosting method in the ROC space for selecting significant features from the features extracted from these images.* Results*. A total of 27 features were obtained from groups A, B, and C. Seven features were selected from groups A and B, while 25 features were selected from groups A and C.* Conclusions*. The selected features in this study were mainly obtained from the root, top, and side areas of the tongue. This is consistent with the tongue partitions employed in traditional Chinese medicine. These results provide scientific evidence to TCM tongue diagnosis for health identification.

## 1. Introduction

As society continues to develop, health status problems have become the main focus of studies in recent years, and health identification has been one of the most important problems. Health identification is a procedure of identifying the condition of a subject as healthy or ill. Western medicine diagnoses a person's health condition based on a series of laboratory examinations. However, these examinations are invasive and time-consuming and require a number of laboratory experiments. As an alternative diagnostic method, traditional Chinese medicine (TCM) proposes Su Wen (*Plain Questions*) as a concept for the preventive treatment of diseases.* Plain Questions* is part of a classical text written during the Zhanguo period of ancient China, which claims that TCM identifies the health status of a person before diagnosing the decease. Health identification is one of the most fundamental diagnostic methods applied in the preventive treatment of diseases in TCM [1]. Compared with Western medicine, TCM uses noninvasive, time-saving methods including tongue and pulse to identify the health status of an individual. In recent years, Western medicine has also begun to focus on establishing preventive treatments for diseases such as health identification, because these results can save medical time, effort, and cost [2].

However, tongue diagnosis in TCM has been criticized due to its subjective diagnostic criteria. Several studies have focused on tongue image diagnosis, and computer image processing has contributed to tongue criteria objectification. Color is the most common feature in tongue diagnosis due to its intuitiveness. The study of Pang et al. introduced lower order moments such as the mean value and standard deviation of color features to diagnose appendicitis [3]. The study of Zhao et al. found color differences between patients with and without chronic hepatitis B [4]. In the current study, tongue coating color features were extracted. For color features, Wang et al. divided tongue colors into 12 categories through the statistical analysis of a large number of tongue images [5]. However, these color features are global and could not describe the local information of the images. The study of Kanawong et al. used color features to classify TCM ZHENG [6]. In the current study, tongue images were divided into several areas, and features in several colored spaces were extracted. The study of Jung et al. preformed a case-control study to investigate color distribution differences in tongue for sleep disorders [7]. The current study also used partition color features. Zhang et al. used their TCM partition knowledge in their AdaBoost algorithm for tongue recognition [8]. The common feature of the above-mentioned studies is that TCM knowledge is used in partitioning. However, from the perspective of TCM, results of these studies were not accepted, because these were based on TCM a priori knowledge. Moreover, these studies focused on disease diagnosis rather than health identification, which is not consistent with TCM diagnosis, in which TCM claims that health identification is more important than disease diagnosis [9].

Zhi et al. used hyperspectral features to classify tongue images [10], and this feature is also a global feature. Yang et al. used texture and curvature features to detect tongue cracks [11, 12]. The current study focused on special tongue images other than tongue diagnosis, and these features are also too global to represent any relationship among these tongue partitions.

Haar-like features are a class of image partition features. Viola and Jones used Haar-like features in face detection [13]. Face detection classifies partitions that contain and do not contain human faces. Currently, this approach has been proven to be successful for face detection. However, its performance significantly declines when applied in other areas [14]. Fu et al. used Haar-like features to segment tongue images from the background [15]. Wang et al. also used Haar-like features to detect and track the lips [16]. However, these two studies did not focus on diagnosing the disease or identifying the health status. Cui et al. used this approach in diagnosing hyperuricemia [17]. However, only a few similar studies can be found.

Feature selection is one of the most important steps in classification and diagnosis. There are three types of feature selection methods: the filter method, which selects an optimal feature set before classification; the wrapper method, which uses a fixed search strategy and interacts with the classifier; and the embedded method, which combines feature selection and the classifier together. Saeys et al. evaluated these methods by applying these in bioinformatics [18].

Boosting algorithm is an effective approach in feather selection and classification. It combines the results of many single classifiers, and the performance of combining these results is better than a single classifier. The reason why this boosting method has a better performance can be explained by the probably approximately correct theory of Valiant [19]. In this theory, the concepts of strong learnable and weak learnable were defined. Schapire was able to prove that these two concepts are equivalent [20]. This conclusion indicates that if a classification model with high predictive accuracy exists, an ensemble of a series of weak models is equivalent to it, even if their predictive results are only slightly better than a random guess.

Viola and Jones used a boosting algorithm in face detection [13] and demonstrated that this algorithm can be employed to cope with both feature selection and classification. However, this algorithm is only suitable for face detection, because the eyes and nose are naturally identifiable. Mamitsuka proposed a boosting algorithm based on the ROC curve for microarray classification [21]. Komori and Eguchi and Long and Servedio also proposed boosting algorithms for maximizing the area under the ROC (AUC) [22, 23]. These studies were able to partly solve the small observation problem but were not used in unbalanced sample problems. In our problem, the number of features is much larger than the number of examples. Fan and Lv proposed a theoretical guarantee for screening features from ultrahigh feature spaces [24].

In order to address this limitation, we propose a ROC-Boosting approach for TCM tongue diagnosis in health identification. This method first screens the features using *t*-test. Then, a Haar-like feature is selected using several different conditions and sends this feature to the ensemble classifier. Our method is generic compared to previous methods, because its conditions include the AUC value, sensitivity, specificity, and their combinations. It can also use positive-negative sample ratio conditions to deal with unbalanced sample problems. We name this method ROC-Boosting, because its feature selection conditions are all relative to the ROC space. Moreover, our method avoids the usage of TCM a priori, and its result is consistent with TCM tongue partitions.

## 2. Subjects and Methods

**Figure 1:**
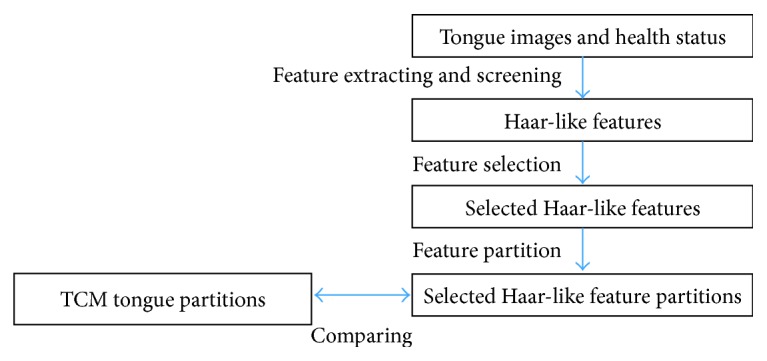
Study procedure. Haar-like features are extracted from tongue images and screened. Then, partitions on the tongue image confirm the TCM diagnosis method.

Tongue images and health information of 1,426 cases from 2011 to 2012 were collected. ROC-Boosting was employed to select Haar-like features extracted from tongue images. Natural partition tongue image features are selected. Then, partitions on the tongue image confirm the TCM diagnosis method. This procedure is illustrated in Figure 1.

### 2.1. Subjects

**Figure 2:**
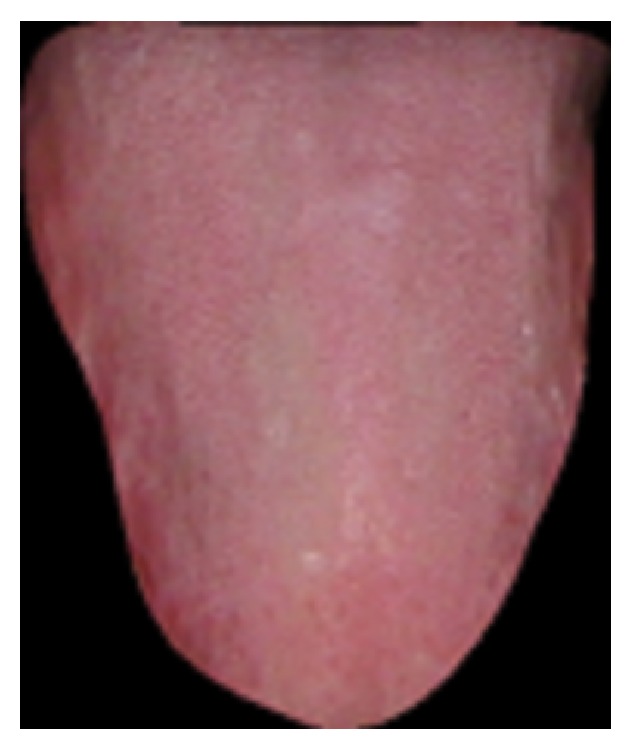
A segmented tongue image. A tongue image segmented from the background to exclude the impact of the background to the feature extraction and selection process.

Tongue images and health information of 1,426 cases from 2011 to 2012 were obtained from the Teaching Hospital of Tianjin University of Traditional Chinese Medicine (TJUTCM). Among these 1,426 cases, 96 cases were excluded due to low quality or duplicate images and health records with missing values. Then, TCM students and experts were employed to discuss the tongue images and health information collected. During this discussion, eight additional duplicate images were found. An outpatient doctor confirmed that these duplicates resulted from the abuse of health insurance ID usage. Hence, these eight images were excluded. Finally, a total of 1,322 cases were included into this study. TCM diagnoses health/illness status before the specific disease, because TCM focuses on the preventive treatment of diseases [8]. For this reason, we focused on the health identification problem in this study, and all subjects were diagnosed as healthy or ill. The 1,322 cases were classified in the following groups: group A, diagnosed as healthy based on both tongue image and health information (*n* = 148 cases); group B, diagnosed as ill based on health information, even if tongue images are normal (TCM considers that tongue changes do not reflect all illnesses, *n* = 332 cases); and group C, diagnosed as ill based on both tongue image and health information (*n* = 842 cases). The number of cases in each group is summarized in Table 1. Before the features were extracted, all images were scaled to 120 × 100 and segmented from the background to exclude the impact of the background to the feature extraction and selection process, as shown in Figure 2.

### 2.2. Methods

#### 2.2.1. Improved Haar-Like Feature Extraction

**Figure 3:**
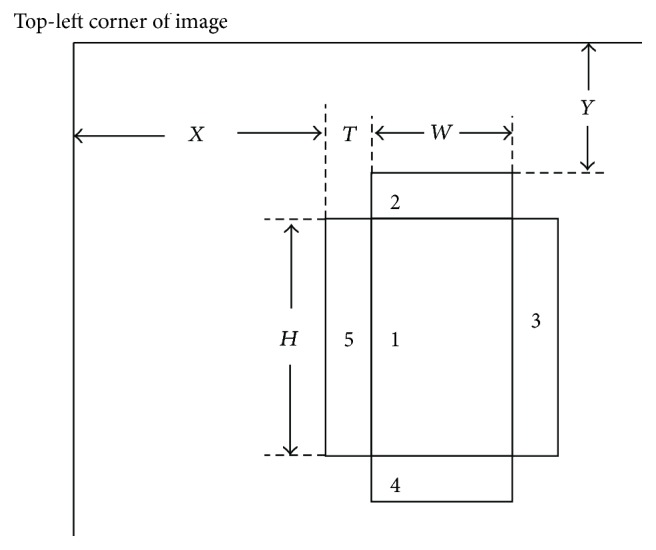
The improved Haar-like feature.

Usually, color features are extracted from the whole tongue and Haar-like features are extracted from partitions. We improved the Haar-like feature to have five partitions, considering that humans focus their view at the center of the target at first glance, as shown in Figure 3. In comparison, the original Haar-like feature was considered as the difference of the sum of the color values between two horizontal or vertical partitions. The center partition of this feature has two parameters: *W* and *H*. These parameters represent the width and height of this partition. The other four surrounding partitions have three parameters: *T*, *X*, and *Y*. *T* represents the width of these four partitions, while *X* and *Y* represent the position of the top-left corner of the Haar-feature. Considering that humans usually focus their view at the center partition and the other four partitions, the number of pixels at the center partition and the other four partitions in the improved Haar-like feature should be equal. To ensure that the number of pixels at the center partition is equal to the other four partitions, *T* is given by [*WH*/(2*W* + 2*H*)], where [ ] represents the maximum integer number smaller than the calculated real number. Parameters *X* and *Y* represent the position of the left-top corner of this feature. The improved Haar-like feature uses the difference between the sum of the pixel color values at the center partition and the other four partitions.

Every improved Haar-like feature is composed of five partitions (1–5). This Haar-like feature has five parameters: *X*, *Y*, *W*, *H*, and *T*. *W* and *H* represent the width and height of the center partition. *T* represents the width of the other partitions. *X* and *Y* represent the position of the Haar-like feature. The feature value can be computed by the difference between the sum of the pixel values at center partition and the other four partitions.

Under this setup, the number of improved Haar-like features is very large. Considering that the number of significant features is very small in our previous study, we reduced the number of features similar to our pervious study [14]. In this study, the density of the parameter grid is reduced to lower the number of improved Haar-like features. We set  *W* ∈ {10, 12, 14,…, 60}, *H* ∈ {10, 12, 14,…, 72}, *X* ∈ {1, 6, 11,…, ⌊100 − *W* − 2*∗T* + 1⌋}, and *Y* ∈ {1, 7, 13,…, ⌊120 − *H* − 2*∗T* + 1⌋} experimentally. After this simplification, the number of features is 98,592 in red, green, and blue color plains, respectively. These features are parts of the inputs of the ROC-Boosting algorithm.

#### 2.2.2. ROC-Boosting

Concerning the difference between improved Haar-like feature values of the healthy and ill population, three tests were designed. The first test investigates the difference between the healthy group (group A) and the ill group diagnosed solely based on health information (group B). Cases in group B were diagnosed as healthy, because differences in tongue images cannot be observed by using the human eye. This test would prove whether a difference exists between these two groups. The second test is designed to verify the difference between the healthy and ill groups (groups A and C), because the difference between these two groups can be observed by using the human eye. The third test is designed to verify the difference between the healthy (A) and ill groups (B and C). As the number of improved Haar-like features becomes very large in comparison to the number of examples, *t*-tests were used to screen the improved Haar-like features in the first instance before applying our method. We reduced the number of improved Haar-like features to approximately 10^4^ through the *P* value of the *t*-test. The *P* value and number of filtered features are listed in Table 2. These features are inputs of our method.

Our method, ROC-Boosting, is illustrated in Algorithm 1. This algorithm calculates the AUC value of all features in every loop. The AUC value would be set to its negative value when the ROC curve is concave; that is, the ROC curve is flipped around the random guess line. This flipping is designed to deal with the reversed prediction feature. Then, ROC-Boosting selects the feature through some conditions, which would be discussed later. After the correctly classified examples and selected features are removed, the loop is restarted. When conditions for selecting features no longer meet, the algorithm stops and presents all selected features. ROC-Boosting selects the most significant features on the tongue images to diagnose subjects from different groups in each step. These features can be used to build classifiers for identifying the health status of subjects from different groups. Verification of conformance between the positions of these features and TCM tongue partitions provides scientific evidence for TCM tongue diagnosis.

As described before, this algorithm is a generic version of the algorithm used by Yang et al. [12]. Viola's method only applies to situations when features with extremely high sensitivity exist such as features that describe the eyes and nose of a human face detection problem. When the condition in step 9 is changed to the highest sensitivity and specificity, ROC-Boosting would be similar to Viola's algorithm. The procedure for running Viola's algorithm has shown that such features do not exist in our problem. This is the reason why we generalized Viola's method.

In our problem, we use the next two conditions in step 9. The first is a negative/positive ratio condition. We compute *r* = *p*/*n* and *r*′ = *p*′/*n*′, where *p* is the number of positive examples, *n* is the number of negative examples, *p*′ is the number of positive examples correctly classified by one feature, and *n*′ is the number of negative examples corrected classified by one feature. This condition is |*r* − *r*′|. The second condition is the AUC |*a* − 0.5| value, where *a* is the AUC of this feature. We used these two conditions, because we did not find any significant feature existing in our problem, and the positive/negative examples are not balanced.

### 2.3. Statistical Analysis Software

We extracted the ROC-Boosting features using a DELL PC (OptiPlex 7020, i5-4590; Quad-Core with 8 GB RAM). The R 2.15.2 64 bit version was the statistical software used [25]. AUC values were calculated using the ROCR package. The code for feature extraction and ROC-Boosting was programmed as a script in R.

## 3. Results and Discussions

### 3.1. Results

For Test 1, only eight features are selected. The ninth feature condition is |*a* − 0.5 | = 0.00943. For Test 2, 25 features are selected. The 26th feature condition is |*a* − 0.5 | = 0.00687. In these two tests, the algorithm comes to its end, because a feature that is better than the guess could no longer be found. For Test 3, 27 features are selected. The 28th feature exceedingly concerns the disease examples than the healthy examples (35/2). The algorithm comes to an end, because features concerning the positive and negative examples could no longer be found.

**Figure 4:**
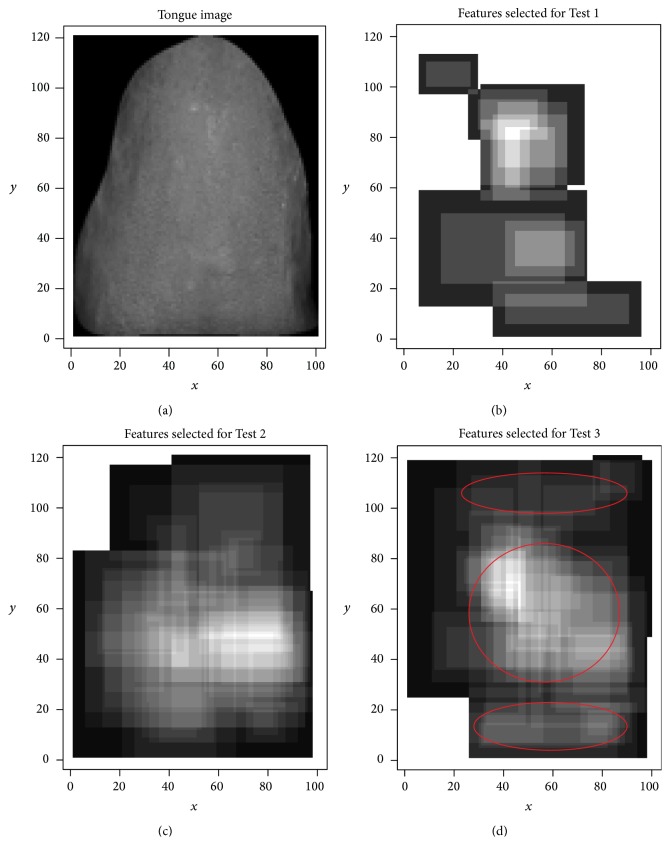
Superposition of all selected features is shown. From (a) to (d):(a) is in grayscale and is used as the background, (b) is the result of Test 1, (c) is the result of Test 2, and (d) is the result of Test 3. In (b, c, d), every rectangle represents one feature. The lighter partition in the rectangle is the center of the feature, and darker partitions surround this feature. Squares at the four corners of the feature were not removed for simplification. When repeatedly overlaid while keeping the color of the darker partitions fixed, the lighter partition would continue to be lighter. Finally, the lightest partition is observed intensively.

We overlaid the selected features and tongue image to investigate the relationship between these two, because we assume that tongue diagnosis is conducted by repetitive observations and each observation corresponds to one feature.  Figure 4 shows the results of the superposition of all selected features. From (a) to (d) in Figure 4: (a) is our sample tongue image; (b) is the superposition of features for Test 1, and its AUC value is 0.662; (c) is for Test 2, and its AUC value is 0.740; and (d) is for Test 3, and its AUC value is 0.723.

Test 1 has a poor performance due to insignificant differences between groups A and B. Even the human eye cannot identify the health status of group B. Test 2 had the best result, because group A is composed of healthy subjects and group C is composed of ill subjects. The shape of the overlaid feature is distributed around the tongue. The difference between these two groups is the most significant among the three tests. The overlaid features for Test 2 are concentrated at the center of the tongue. The overlaid features in the last figure consist of three areas: root, center, and top of the tongue image. We marked these three areas in the last figure. Its performance is slightly worse than Test 2 due to the interference of group B.

### 3.2. Discussions

Our method is more generic than previous studies. Viola's method is only applicable to situations where high sensitivity features exist [13]. In this situation, the algorithm selects features with increasing specificity from high sensitivity features. A high performance classifier would be built when high sensitivity is maintained and specificity is increased. ROC-Boosting can also use specificity and sensitivity simultaneously, and the value of specificity and sensitivity can be relatively low. It can also use other conditions to select features. ROC-Boosting also works well even when high sensitivity features do not exist. One of the problems of health identification is the use of Haar-like features on tongue images. In a preexperiment, we tested all features in this study. No high sensitivity feature exists in our data, and we confirmed that Viola's method works. ROC-Boosting was able to select the features.

Learnability theory guarantees the effectiveness of the ROC-Boosting algorithm. If a high performance classifier exists in a health identification problem using Haar-like features on the tongue image, which is the basic hypothesis of this study, an ensemble of weak classifiers whose performance is better than the random guess would be equivalent to it. In ROC-Boosting, the condition |*a* − 0.5 | >0 keeps every weak classifier better than the random guess.

**Figure 5:**
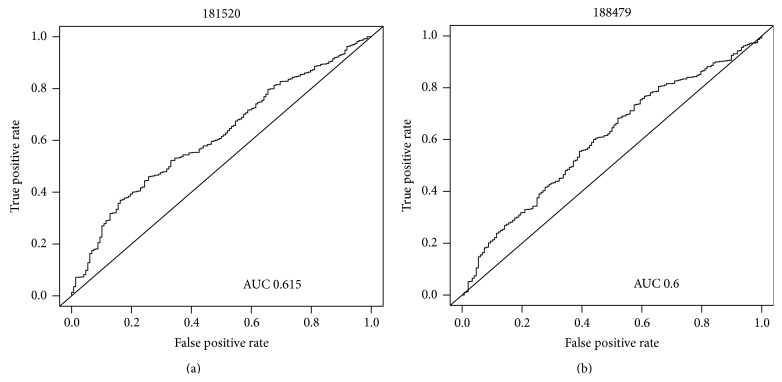
Two features of the AUC value larger than 0.5 are shown. Feature 181,520 in the left is selected, because the whole ROC curve is laid upward the random guess line. Feature 188,479 in the right is excluded, because its ROC curve crosses the random guess line at the corners of the ROC space.

Furthermore, weak classifiers should focus on both negative and positive examples. As shown in Figure 5, even though these two features (181,520 in the left subfigure and 188,479 in the right subfigure) were |*a* − 0.5 | >0, 188,479 is excluded, because it focuses on the positive example only. In ROC-Boosting, |*r* − *r*′| excludes these features.

**Figure 6:**
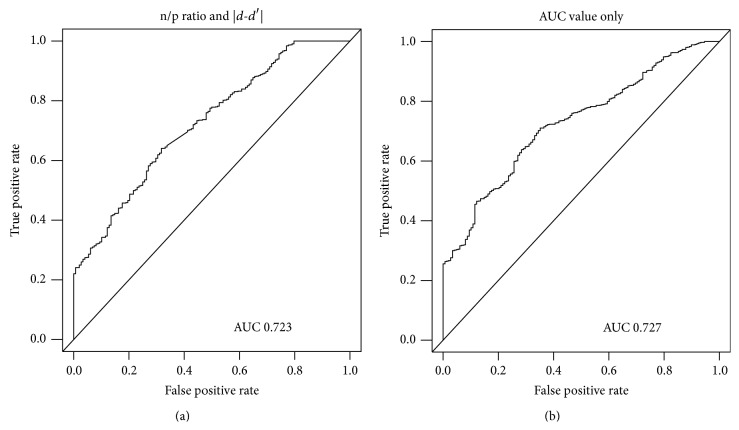
Comparative results of the negative/positive ratio and |*d* − *d*′| conditions, as well as the solely AUC value condition. Although the AUC value on the right image (0.727) is slightly larger than the left image (0.723), the solely AUC value condition obtains this superiority, because it is inclined to predict all subjects as with disease. When the number of positive and negative subjects is unbalanced, prediction tending to the major class is the most common phenomenon.

We also compared the two different conditions of ROC-Boosting for Test 3. The first condition is the negative/positive ratio and the |*d* − *d*′| condition, which was used in our study. The second condition is the solely AUC value condition, which was proposed in previous studies. Comparative results are shown in Figure 6. We also selected 27 features using the solely AUC value condition. However, after the 16th feature was selected, the next features selected by this condition could only correctly predict one to four subjects at two ends of the feature value. The number of correctly predicted subjects determines that the AUC value increases in this step. This indicates that the AUC value is unsustainable after the 16th feature is selected. To compare the results of two different conditions, we run the program continuously. Although the AUC value of the right image (0.727) is slightly larger than the left image (0.723), the solely AUC condition obtains this superiority, because it is inclined to predict all subjects as with disease. When the number of positive and negative subjects is unbalanced, prediction tending to the major class is the most common phenomenon. Relatively, the negative/positive ratio and |*d* − *d*′| condition are associated with both positive and negative classes, while obtaining a similar AUC value. The correct prediction of both healthy and ill subjects is equally important in health identification.

## 4. Conclusion

We propose the application of the ROC-Boosting algorithm for health identification. This algorithm uses filtered Haar-like features and selects features from both positive and negative examples. The features selected for diagnosing health and ill subjects are concentrated in the root, center, and top partitions of the tongue images. Unlike previous studies, these partitions are not results of preexperience. A deterministic algorithm presents these partitions. These results provide scientific evidence to TCM tongue diagnosis for health identification.

## Figures and Tables

**Algorithm 1:**
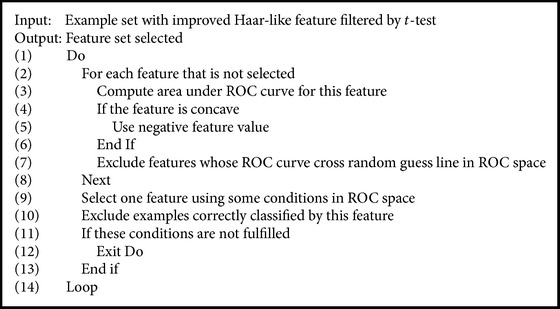
ROC-Boosting algorithm.

**Table 1:** Summary of the number of cases in each group.

Group	Status	Number of cases
A	Healthy, diagnosed based on tongue image and heath information	148
B	Ill, diagnosed based on health information, but tongue image is normal	332
C	Ill, diagnosed based on both tongue image and health information	842
Total		1,322

**Table 2:** *P* values and the number of features between groups.

Test	Group	*P* value	Number of filtered features
1	A/B	0.05	14,878
2	A/C	0.00005	12,280
3	A/B + C	0.0005	11,260
